# *GLIS1*, a potential candidate gene affect fat deposition in sheep tail

**DOI:** 10.1007/s11033-021-06468-w

**Published:** 2021-06-16

**Authors:** Rongsong Luo, Xiaoran Zhang, Likai Wang, Li Zhang, Guangpeng Li, Zhong Zheng

**Affiliations:** 1 State Key Laboratory of Reproductive Regulation & Breeding of Grassland Livestock, School of Life Sciences, Inner Mongolia University, Hohhot, 010070 China; 2 State Key Laboratory of Genetic Resources and Evolution, Kunming Institute of Zoology, Chinese Academy of Sciences, Kunming, 650223 China; 3 State Key Laboratory for Agrobiotechnology, College of Biological Sciences, China Agricultural University, Beijing, 100193 China

**Keywords:** Population Genomics, Fat tail, Mongolian sheep, *GLIS1*, *PDGFD*

## Abstract

Fat deposition in sheep tails is as a result of a complicated mechanism. Mongolian sheep (MG) and Small Tail Han sheep (STH) are two fat-tailed Chinese indigenous sheep breeds while DairyMeade and East Friesian (DS) are two thin-tailed dairy sheep breeds recently introduced to China. In this study, population genomics analysis was applied to identify candidate genes associated with sheep tails based on an in-depth whole-genome sequencing of MG, STH and DS. The selective signature analysis demonstrated that *GLIS1*, *LOC101117953*, *PDGFD* and *T* were in the significant divergent regions between DS and STH–MG. A nonsynonymous point mutation (g.27807636G>T) was found within *GLIS1* in STH–MG and resulted in a Pro to Thr substitution. As a pro-adipogenic factor, *GLIS1* may play critical roles in the mesodermal cell differentiation during fetal development affecting fat deposition in sheep tails. This study gives a new insight into the genetic basis of species-specific traits of sheep tails.

**Supplementary Information:**

The online version contains supplementary material available at 10.1007/s11033-021-06468-w.

## Introduction

Sheep (*Ovis aries*) was one of the first domesticated livestock, providing humans with meat, milk, fur, and wool products. Domestication, natural and artificial selection have resulted in remarkable phenotypic diversity in animal appearance, growth rate, local adaptation, and fertility rate [1]. China has diverse landscapes and climatic features. Indigenous sheep breeds have developed good adaptation to various environmental conditions, such as harsh winter, drought, food scarcity, and high altitude, to become essential livestock in the animal husbandry industry [2]. These breeds with different traits adapted in various production systems in the vast geographical regions of China provides us opportunities to study the genetic basis of adaptation.

The wild ancestors of domestic sheep had thin tails. The fat tails in sheep are perceived to have developed following domestication as an adaptive response to store energy for use during migration and in harsh winter [3]. In china, the indigenous fat-tailed sheep breeds mainly originated from Mongolian sheep. They are widely distributed in northern China and Mongolian People's Republic. The over-deposition of fat in the tails helps the sheep to overcome harsh environments such as extreme cold, drought, and food scarcity but may also compromise reproduction and fattening and reduce economic value of sheep rearing [4, 5]. However, the fat-tailed sheep provide us an ideal model for studying the mechanism of fat deposition in animals. In recent years, population genomics has been extensively and effectively applied to identify candidate genes associated with phenotypic diversity and important agronomic traits in domestic animals. Previous studies provided evidence of promising candidate genes influencing tail types based on single nucleotide polymorphism (SNP) markers [3, 6–8]. However, the fat-tailed trait may be a contribution of multiple genes and have a complicated co-regulation mechanism [9–11]. DairyMeade and East Friesian are the two dairy breeds recently introduced to China with large frame, fast growth rate, lean carcass and typical thin tails. Moreover, DairyMeade is a new dairy sheep breed developed in New Zealand and originated from East Friesian [12, 13]. These two breeds provide us new materials to study the mechanism of fat deposition in sheep tails. In this study, we conducted an in-depth whole-genome sequencing of two typical fat-tailed breeds (Mongolian and Small Tail Han sheep) and two typical thin-tailed breeds (DairyMeade and East Friesian sheep) and provided new insights into the genetic basis of species-specific adaptive traits of the fat tail.

## Materials and methods

### Sampling, DNA extraction, and sequencing

Ear tissues of 13 dairy sheep (including 9 DairyMeade from 3 different pedigrees, 2 East Friesian from 2 different pedigrees, 1 East Friesian × Small Tail Han sheep F_1_ and 1 DairyMeade × F_1_ F_2_ sheep), 7 Small Tail Han sheep from 7 different pedigrees, and 9 Mongolian sheep from 9 different pedigrees were collected from different locations in Inner Mongolia Autonomous Region, China, for whole-genome resequencing (Fig. S1; Table S1). All the ear tissues collected were immediately stored in liquid nitrogen. The animal experimental procedures were performed according to the guidelines set by the Ethics Committee of Inner Mongolia University (IMU-sheep-2018-011).

Genomic DNA was extracted from the ear tissues using the standard phenol–chloroform method. The quality and quantity of the DNA were determined using a Qubit 2.0 fluorometer (Invitrogen). Next-generation sequence library construction was performed with 3 μg of genomic DNA according to the standard Illumina library preparation protocols and insert size of 350 bp. All libraries were sequenced on an Illumina Hiseq 2500 platform to generate paired-end reads. The resequencing depth ranged from 12.3 × to 35.5 × fold coverage, with an average depth of 18.14 × .

### Reads mapping and SNP calling

The adaptors and low-quality sequences of raw reads were trimmed and filtered to obtain clean reads using FastQC (version 0.11.7) [14] and Trimmomatic (version 0.36) [15]. High-quality paired-end reads were mapped to the sheep reference genome OAR4.0 using the BWA-MEM alignment tool [16] implemented in BWA software with the command 'mem -t 10 -M'. Duplicated reads were removed following the alignment of bam files using the SORTSAM and MARKDUPLICATES function in the PICARDS package (picard-tools-2.18, http://picard.sourceforge.net). SAMTOOLS [17] was used to create bam files index. The SNPs were then called using bcftools (mpileup) and filtered by vcftools (-minQ 30 --min-alleles 2 --max-alleles 2 --min-meanDP 4.0 --max-meanDP 72.0 --max-missing 0.9 --non-ref-ac 2 --remove-indels --recode --recode-INFO-all) [18]. Finally, all SNPs were annotated with ANNOVAR [19] according to NCBI’s gene annotation database.

### Population structure and genomic diversity analysis

Based on the autosomal genetic variants, PLINK v1.9 [20] was used to calculate the individual genetic distances of the sheep. MEGA v7.0 [21] was then used to construct the Neighbor-Joining (NJ) tree for the genetic distance matrix. The fourfold degenerate sites were also used to build ML and NJ trees. The principal component analysis in all sheep was conducted using vcftools and PLINK with the parameters ‘--maf 0.05 --max-missing 0.9 --chr-set 26’. The nucleotide diversity (π) was calculated using vcftools with the parameter ‘--window-pi50000 --window-pi-step 25000’. The PopLDdecay software [22] was used to calculate r^2^ (-minMAF 0.05 -hwcutoff 0.001 -Het 0.88 -Miss 0.25) for the pairs of SNPs and to plot the LD curves. To remove the bias introduced by differing sample sizes in different populations, individuals in each population were randomly sampled to maintain a consistent sample size during the calculations (7 individuals per group). Only SNPs with a minor allele frequency (MAF) greater than 0.05 were considered.

### Genomic selective sweep analysis

Selective sweep signals were identified using the population differentiation index (${F}_{\mathrm{ST}}$, the DS group vs. the STH and MG groups) and locus-specific branch lengths, LSBL [23, 24] based on the sliding window strategy (window size 50 kb; step size 25 kb). LSBL was estimated based on pairwise ${F}_{\mathrm{ST}}$ values [25] of each polymorphic site from three groups: Target (DS), Control (STH), and Background (MG). The formula LSBL = (${F}_{\mathrm{ST}}$(DS–STH) + ${F}_{\mathrm{ST}}$(DS–MG) − ${F}_{\mathrm{ST}}$(STH–MG))/2 was used. The threshold for identifying the putative selection regions in the ${F}_{\mathrm{ST}}$ and LSBL analyses was empirically set at the top 1% percentile outliers. The putative genes under selection were submitted to DAVID [26] for the Kyoto Encyclopedia of Genes and Genomes (KEGG) enrichment analysis. Fisher’s Exact Test was used for *p*-value correction. Only terms with a *p*-value less than 0.05 were considered significant and listed. Data analysis and visualization were carried out with customized R scripts.

## Results

### Population structure and genomic diversity

Whole-genome sequencing was carried out at an average depth of 18.14 × coverage (Table S2), on the ear tissues collected from sheep in different regions of Inner Mongolia, China. After rigorously filtering, a total of 25,375,422 high-quality SNPs were obtained for further analysis. Among them, 15,525,859 SNPs were in intergenic regions, while 171,462 SNPs were in exonic regions (Table S3). The genetic relationships between the sheep breeds were explored based on all the genetic variants and fourfold degenerate sites. The phylogenetic tree constructed by the neighbor-joining (NJ) method showed that each breed population had a distinct clade (DairyMeade sheep and East Friesian sheep, DS; Small Tail Han sheep, STH; Mongolia sheep, MG) (Fig. S2a). Similar genetic affinities were obtained in phylogenetic trees constructed by neighbor-joining (Fig. S3a) and maximum-likelihood (ML) methods (Fig. S3b) using fourfold degenerate sites. Principle component analysis (PCA) also uncovered different population structuring among DS, MG and STH, and the PC1 (4.06%) revealed the fat-tailed and thin-tailed sheep variants (Fig. S2b). ADMIXTURE analysis revealed that the fat-tailed and thin-tailed sheep, belonged to different clades (*K* = 2), and there was no genetic exchange (Fig. S4).

The genetic diversity index was calculated based on the whole-genome genetic variants. Compared with STH and MG, DS showed a lower nucleotide diversity (DS, π = 2.533e−3; STH, π = 2.79e−3; MG, π = 2.87e−3) (Figs. S2c, S5) and a slower decay rate of linkage disequilibrium (LD, (dropped to half of its maximum at 79 kb, STH group (62 kb) and MG group (46 kb)) (Fig. S2d). These results suggest that indigenous breeds (MG and STH) have a higher genetic diversity, while bottlenecking and/or inbreeding occurred in the two dairy sheep breeds.

### Selective signatures in fat- and thin-tailed sheep

**Fig. 1:**
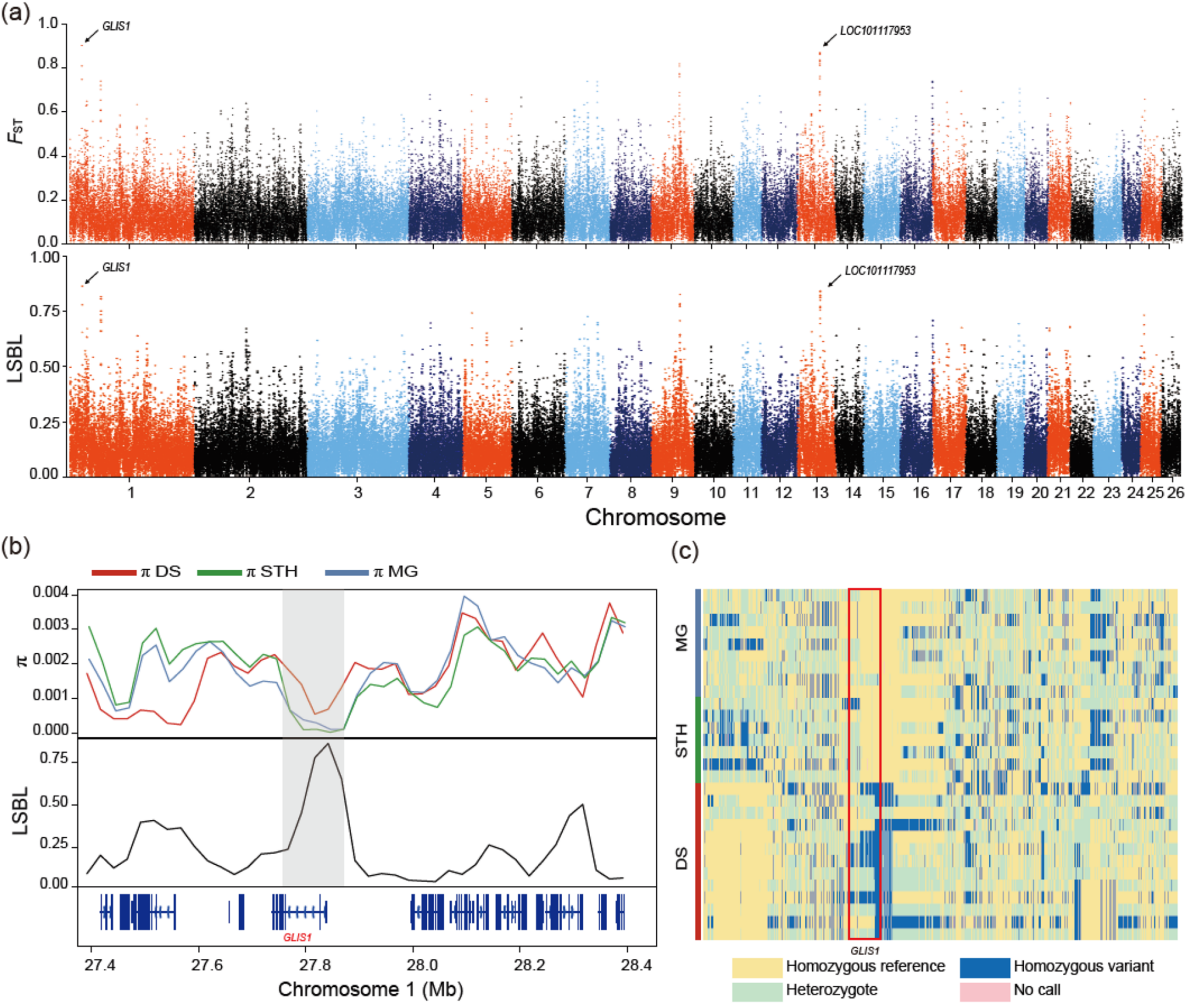
Selective-sweep analysis by comparing genomes between thin-tailed DS (dairy sheep, DairyMeade and East Friesian) and fat-tailed STH–MG (Small Tail Han sheep and Mongolian sheep). (a) Distribution of population differentiation index (${F}_{\mathrm{ST}}$, top panel) and the lineage-specific branch length (LSBL, bottom panel) between DS and STH–MG in a 50 kb sliding window with a 25 kb step increment across all autosomes. (b) π and LSBL values around the genomic region on chromosome 1 (from 27.4 to 28.4 Mb) between DS and STH–MG populations. *GLIS1* is located in this genomic sweep region. The red, green and blue cells represent DS, STH and MG population, respectively. (c) Haplotype pattern of the selective-sweep region. Haplotype pattern in a region defined by SNPs that are at a high frequency in DS and at a low frequency in STH–MG. Each column is a polymorphic genomic location, each row is a phased haplotype, and the colored column on the left denotes the population identity of the individuals. The reference/alternative allele is indicated in light yellow/green. (Color figure online)

Tail size was the prominent phenotypic difference between DS and MG/STH. We analyzed the inter/intra-population diversities of the highly significant sweep regions to explore the genetic basis underlying fat deposition in the tail. The population differentiation index ${F}_{\mathrm{ST}}$ and the LSBL of DS, STH and MG, was calculated on a sliding-window basis (50 kb sliding window with 25 kb step increment) to detect the candidate divergent regions. A total of 798 genomic regions were shown to have increased differentiation index between DS and STH–MG (${F}_{\mathrm{ST}}$> 0.42; LSBL > 0.435; both were at the top 1% threshold) (Fig. 1a; Table S4). In total, 510 shared protein-coding genes (619 and 614 genes were identified by ${F}_{\mathrm{ST}}$ and LSBL, respectively) were identified with signatures of selection (Table S5), which accounted for 1.96% of the whole-genome annotated genes (a total of 26,076). The functional enrichment analysis (in terms of KEGG) for the detected selective genes revealed that overrepresented functional categories were associated with cell growth and immunity, such as focal adhesion (adjusted *p*-value = 0.00086) and T cell receptor signaling pathway (adjusted *p*-value = 0.0013) (Table S6).

**Fig. 2:**
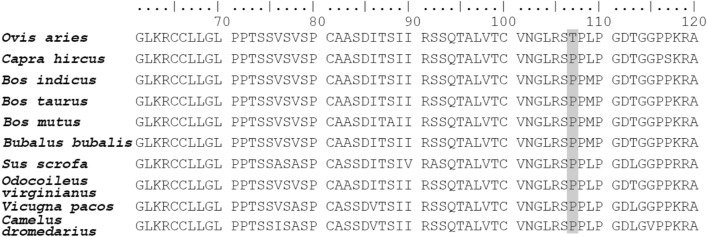
Alignment of the amino acid sequences of *GLIS1* protein in different mammals. Positions in which the amino acids differ are highlighted in grey

Amongst the candidate divergent regions, two putative sweeps showed the highest population differentiation scores. One was located on chromosome 1 (LSBL = 0.86 and ${F}_{\mathrm{ST}}$ = 0.79) as displayed in the Manhattan plots (Fig. 1a). This region, from 27.75 to 27.86 Mb, only harbors the *GLIS1* gene (Fig. 1b). Further haplotype analysis showed that the haplotype pattern in DS was strikingly different from STH and MG (Figs. 1c, S6). A nonsynonymous point mutation (g.27807636G>T) found within *GLIS1* in STH–MG resulted in a nonsynonymous Pro107 → Thr (P107T) substitution, thus making STH–MG different from DM and other thin tail mammals in this locus (Fig. 2). The second putative sweep appeared in a locus on chromosome 13 (LSBL = 0.82 and ${F}_{\mathrm{ST}}$ = 0.78) harboring three pseudogenes, including *LOC101117953*, *LOC101118207* and *LOC101110166* (Fig. S7). Another genomic region (from 3.825 to 3.90 Mb) on chromosome 15 also exhibited strong selection signatures (LSBL = 0.92, 0.93) between DS and STH–MG (Fig. S8), that harbors *PDGFD* gene, a member of the platelet-derived growth factor family. Other genes related to sheep tail traits, such as *T* (LBSL = 1.02, ${F}_{\mathrm{ST}}$ = 0.53) were also found in this study.

## Discussion

Dairy sheep breeds with typical thin tail phenotype, including DairyMeade and East Friesian, are recently used to hybrid with fat-tailed Mongolian sheep and Small Tail Han sheep to create new dairy sheep breed in Inner Mongolia, China. To our surprise, all the F_1_ sheep have significantly thinner tail than Mongolian sheep or Small Tail Han sheep, without segregation of character. Since these two dairy sheep breeds were not used in any studies related to tail fat deposition before, we then collect all the available pedigrees of DairyMeade and East Friesian in China and compared their genome with local fat-tailed Mongolian sheep and Small Tail Han sheep.

The fat tail phenotype in sheep occurs as a result of multiple genes. With the usage of new genomic materials, this study revealed that there was a recent selective sweep at *GLIS1* locus in the ovine genome. *GLIS1* is a zinc finger protein that acts as both an activator and repressor of transcription [27]. In mouse embryonic development, *GLIS1* starts to express in the forelimb, hindlimb and tail at 10.0 days post coitus (dpc), then it expresses in the anterior region of the forelimb, ventral part of the body and tail at 10.5 dpc and the expression is increased at 11.0 dpc, which is consistent with mesoderm differentiation [28]. In a recent study, *GLIS1* was recognized as a novel pro-adipogenic transcription factor. It is highly expressed in bipotent muscle satellite cells. But when overexpressed, increased occupancy of *GLIS1* is observed at the promoters of adipogenic genes *Adipoq*, *Cebpa* and *Ucp1*, and drives brown adipogenesis both in vitro and in vivo [29]. However, *GLIS1* role in sheep has not been extensively studied. A SNP in *GLIS1* affects the feed efficiency in Dual Purpose and Blackface rams [30], which may also be related to different muscle and fat ratio in the carcass. DS and MG/STH had remarkable differences in growth rate and tail phenotype. In both newborn and adult DS, almost no fat was deposited in the tail. However, MG and STH, had a large amount of fat deposited to the ventral region of the tail and subcutaneous tissue. It is worth noting that fat deposition in the ventral region of the tail was observed as early as in the postnatal stage, indicating that the tail phenotype is determined during fetal development. Thus, it could be an innate feature of adaptation for MG and STH to face the challenges of cold and food scarcity lambing season (March to April) in northern China. Combined with the information together, we hypothesized that, *GLIS1*, as a pro-adipogenic factor, plays a key role in mesodermal cell differentiation during fetal development in fat-tailed sheep to initiate differentiation of preadipocytes and fat accumulation.

Previous studies reported that *LOC101117953* and *BMP2* (bone morphogenetic protein 2, from 48,387,181 to 48,400,679 bp on chromosome 13) were related to tail-fat deposition [6–8]. *LOC101117953* is a retro-copy of *PPP1CC* (protein phosphatase PP1-gamma catalytic subunit gamma), which is not expressed in adult tissues as it lacks promoter region, and is thus less likely to be the causative gene for the tail phenotypes [8]. Previous studies also revealed that *PDGFD* is a likely causal gene for fat deposition in sheep tail, which promotes proliferation and inhibits differentiation of preadipocyte [11, 31–33]. Two SNPs in *PDGFD* significantly affect the tail length and width [34]. *T*, a key regulator of mesoderm formation during early development, was found related to short-tail phenotype in Hulunbuir sheep, a subpopulation of Mongolia sheep [35]. It may also be related to the caudal vertebra phenotype differences between DS and STH/MG, as DS has long straight tails while STH/MG has relatively shorter tails with a slightly curved tip.

This study revealed that the ovine genome has recently encountered a selective sweep at *GLIS1* locus. As a novel pro-adipogenic transcription factor, *GLIS1* may initiate the accumulation and differentiation of preadipocytes in the tails during fetal development and affect the tail phenotypes in sheep.

## Conclusions

Fat tail in sheep is occurs as a result of multiple genes. This study demonstrated that *GLIS1*, *LOC101117953*, *PDGFD* and *T* have encountered a recent selective sweep. A nonsynonymous point mutation (g.27807636G>T) within *GLIS1* locus in STH–MG resulted in a Pro to Thr substitution. As a pro-adipogenic factor, *GLIS1* may play critical roles in the mesodermal cell differentiation during fetal development and affect fat deposition in sheep tails. This study gives a new insight into the genetic basis of species-specific adaptive traits in sheep and provides a novel opportunity to develop therapies for complex diseases related to fat metabolism.

## Supplementary Information

Below is the link to the electronic supplementary material.

Supplementary file1 (DOCX 1511 kb)

Supplementary file2 (XLSX 98 kb)

## Data Availability

The whole-genome resequencing datasets used in this study were submitted to the National Center for Biotechnology Information (NCBI) Sequence Read Archive with the accession code PRJNA531155. Additional information could be found in Supplementary Materials.

## References

[1] Sabeti PC, Varilly P, Fry B, Lohmueller J, Hostetter E, Cotsapas C, Xie X (2007). Genome-wide detection and characterization of positive selection in human populations. Nature.

[2] Yang J, Li W, Lv F, He S, Tian S, Peng W, Sun Y, Zhao Y, Tu X, Zhang M, Xie X, Wang Y, Li J, Liu Y, Shen Z, Wang F, Liu G, Lu H, Kantanen J, Han J, Li M, Liu M (2016). Whole-genome sequencing of native sheep provides insights into rapid adaptations to extreme environments. Mol Biol Evol.

[3] Moradi MH, Nejati-Javaremi A, Moradi-Shahrbabak M, Dodds KG, McEwan JC (2012). Genomic scan of selective sweeps in thin and fat tail sheep breeds for identifying of candidate regions associated with fat deposition. BMC Genet.

[4] Kilminster TF, Greeff JC (2011). A note on the reproductive performance of Damara, Dorper and Merino sheep under optimum management and nutrition for Merino ewes in the eastern wheatbelt of Western Australia. Trop Anim Health Prod.

[5] Frisch RE (1987). Body fat, menarche, fitness and fertility. Hum Reprod.

[6] Moioli B, Pilla F, Ciani E (2015). Signatures of selection identify loci associated with fat tail in sheep. J Anim Sci.

[7] Wei C, Wang H, Liu G, Wu M, Cao J, Liu Z, Liu R, Zhao F, Zhang L, Lu J, Liu C, Du L (2015). Genome-wide analysis reveals population structure and selection in Chinese indigenous sheep breeds. BMC Genomics.

[8] Pan Z, Li S, Liu Q, Wang Z, Zhou Z, Di R, An X, Miao B, Wang X, Hu W, Guo X, Lv S, Li F, Ding G, Chu M, Li Y (2019). Rapid evolution of a retro-transposable hotspot of ovine genome underlies the alteration of BMP2 expression and development of fat tails. BMC Genomics.

[9] Zhao F, Deng T, Shi L, Wang W, Zhang Q, Du L, Wang L (2020). Genomic scan for selection signature reveals fat deposition in Chinese indigenous sheep with extreme tail types. Animals.

[10] Xu SS, Ren X, Yang GL, Xie XL, Zhao YX, Zhang M, Shen ZQ, Ren YL, Gao L, Shen M, Kantanen J, Li MH (2017). Genome-wide association analysis identifies the genetic basis of fat deposition in the tails of sheep (*Ovis aries*). Anim Genet.

[11] Dong K, Yang M, Han J, Ma Q, Han J, Song Z, Luosang C, Gorkhali NA, Yang B, He X, Ma Y, Jiang L (2020). Genomic analysis of worldwide sheep breeds reveals PDGFD as a major target of fat-tail selection in sheep. BMC Genomics.

[12] ME K, JE K, CB P (2014) Sheep dairying in New Zealand—the Kingsmeade story. Proc N Z Soc Anim Prod 74:58–61

[13] Allison AJ (1995). Importing a sheep which offers more—the East Friesian. Proc N Z Soc Anim Prod.

[14] Bioinformatics B (2011). FastQC: a quality control tool for high throughput sequence data.

[15] Bolger AM, Lohse M, Usadel B (2014). Trimmomatic: a flexible trimmer for Illumina sequence data. Bioinformatics.

[16] Li H (2013) Aligning sequence reads, clone sequences and assembly contigs with BWA-MEM. arXiv preprint arXiv:1303.3997

[17] Li H, Handsaker B, Wysoker A, Fennell T, Ruan J, Homer N, Marth G, Abecasis G, Durbin R (2009). The sequence alignment/map format and SAMTOOLS. Bioinformatics.

[18] Li H (2011). A statistical framework for SNP calling, mutation discovery, association mapping and population genetical parameter estimation from sequencing data. Bioinformatics.

[19] Wang K, Li M, Hakonarson H (2010). ANNOVAR: functional annotation of genetic variants from high-throughput sequencing data. Nucleic Acids Res.

[20] Purcell S, Neale B, Todd-Brown K, Thomas L, Ferreira MA, Bender D, Maller J, Sklar P, De Bakker PI, Daly MJ (2007). PLINK: a tool set for whole-genome association and population-based linkage analyses. Am J Hum Genet.

[21] Kumar S, Stecher G, Tamura K (2016). MEGA7: molecular evolutionary genetics analysis version 7.0 for bigger datasets. Mol Biol Evol.

[22] Zhang C, Dong S, Xu J, He W, Yang T (2018). PopLDdecay: a fast and effective tool for linkage disequilibrium decay analysis based on variant call format files. Bioinformatics.

[23] Ai H, Fang X, Yang B, Huang Z, Chen H, Mao L, Zhang F, Zhang L, Cui L, He W (2015). Adaptation and possible ancient interspecies introgression in pigs identified by whole-genome sequencing. Nat Genet.

[24] Shriver MD, Kennedy GC, Parra EJ, Lawson HA, Sonpar V, Huang J, Akey JM, Jones KW (2004). The genomic distribution of population substructure in four populations using 8,525 autosomal SNPs. Hum Genomics.

[25] Akey JM, Zhang G, Zhang K, Jin L, Shriver MD (2002). Interrogating a high-density SNP map for signatures of natural selection. Genome Res.

[26] Sherman BT, Lempicki RA (2009). Systematic and integrative analysis of large gene lists using DAVID bioinformatics resources. Nat Protoc.

[27] Kim Y, Lewandoski M, Perantoni AO, Kurebayashi S, Nakanishi G, Jetten AM (2002). Identification of Glis1, a novel Gli-related, Krüppel-like zinc finger protein containing transactivation and repressor functions. J Biol Chem.

[28] Nakashima M, Tanese N, Ito M, Auerbach W, Bai C, Furukawa T, Toyono T, Akamine A, Joyner AL (2002). A novel gene, GliH1, with homology to the Gli zinc finger domain not required for mouse development. Mech Dev.

[29] Tosic M, Allen A, Willmann D, Lepper C, Kim J, Duteil D, Schüle R (2018). Lsd1 regulates skeletal muscle regeneration and directs the fate of satellite cells. Nat Commun.

[30] Cockrum RR, Pickering NK, Anderson RM, Hyndman DL, Bixley MJ, Dodds KG, Stobart RH, McEwan JC, Cammack KM (2012) Identification of single nucleotide polymorphisms associated with feed efficiency in RAMS. In: Conference, vol 79. Western Section American Society of Animal Science

[31] Zhao F, Deng T, Shi L, Wang W, Zhang Q, Du L, Wang L (2020). Genomic scan for selection signature reveals fat deposition in Chinese Indigenous sheep with extreme tail types. Animals (Basel).

[32] Li X, Yang J, Shen M, Xie X, Liu G, Xu Y, Lv F, Yang H, Yang Y, Liu C, Zhou P, Wan P, Zhang Y, Gao L, Yang J, Pi W, Ren Y, Shen Z, Wang F, Deng J, Xu S, Salehian-Dehkordi H, Hehua E, Esmailizadeh A, Dehghani-Qanatqestani M, Štěpánek O, Weimann C, Erhardt G, Amane A, Mwacharo JM, Han J, Hanotte O, Lenstra JA, Kantanen J, Coltman DW, Kijas JW, Bruford MW, Periasamy K, Wang X, Li M (2020). Whole-genome resequencing of wild and domestic sheep identifies genes associated with morphological and agronomic traits. Nat Commun.

[33] Wei C, Wang H, Liu G, Wu M, Cao J, Liu Z, Liu R, Zhao F, Zhang L, Lu J, Liu C, Du L (2015). Genome-wide analysis reveals population structure and selection in Chinese indigenous sheep breeds. BMC Genomics.

[34] Li Q, Lu Z, Jin M, Fei X, Quan K, Liu Y, Ma L, Chu M, Wang H, Wei C (2020). Verification and analysis of sheep tail type-associated PDGF-D gene polymorphisms. Animals.

[35] Zhi D, Da L, Liu M, Cheng C, Zhang Y, Wang X, Li X, Tian Z, Yang Y, He T, Long X, Wei W, Cao G (2018). Whole genome sequencing of Hulunbuir short-tailed sheep for identifying candidate genes related to the short-tail phenotype. G3 (Bethesda).

