# Supplementary material for: GLIS1, a potential candidate gene affect fat deposition in sheep tail
Source: Mol Biol Rep. 2021 Jun 16;48(5):4925–31. doi: 10.1007/s11033-021-06468-w (PMC8260413; doi:10.1007/s11033-021-06468-w)
Supplement: Supplementary file 1 — Supplementary file1 (DOCX 1511 kb) [file 11033_2021_6468_MOESM1_ESM.docx]

## Supplementary Figures


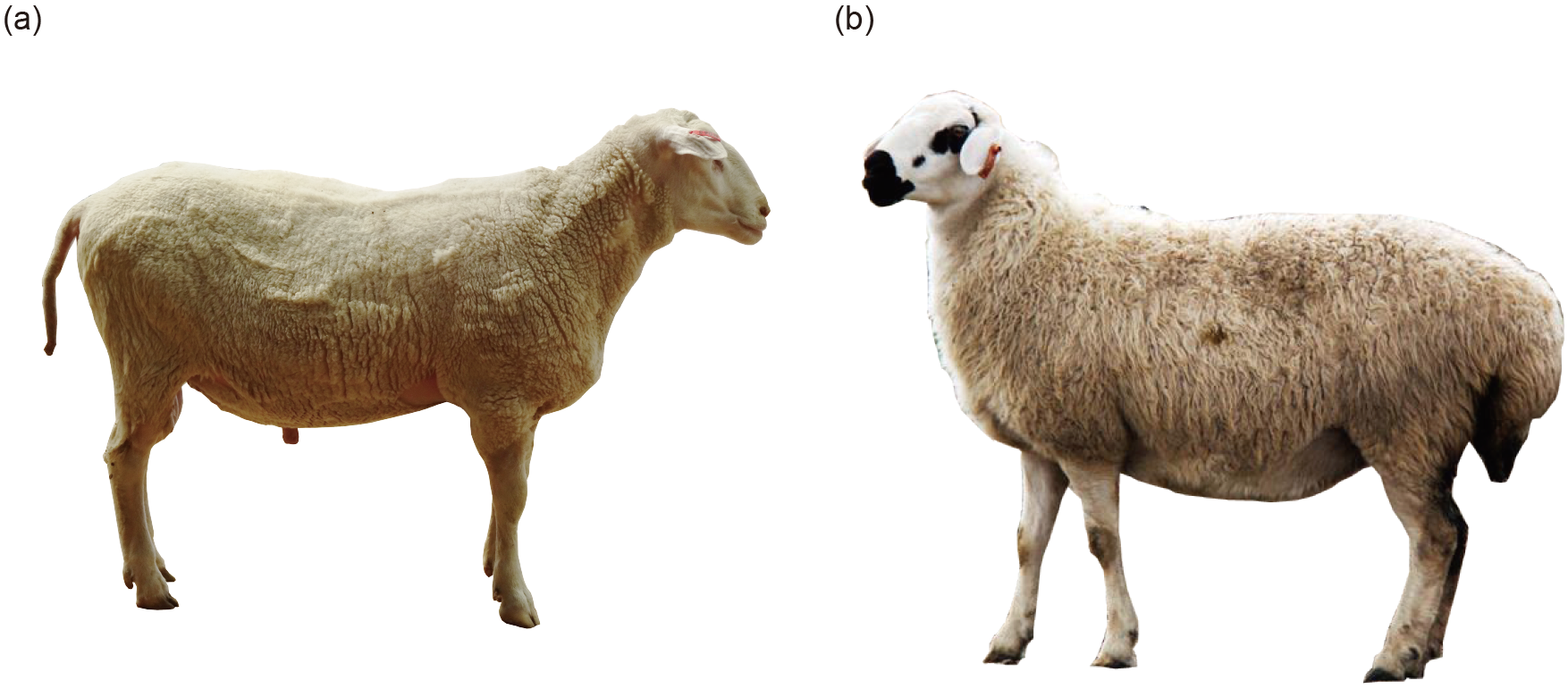


**Fig. S1** Representative picture of the tail phenotype of DairyMeade sheep (a) and Mongolia sheep (b). DairyMeade has a typical thin tail without any fat deposition, while Mongolia sheep has a typical fat tail.


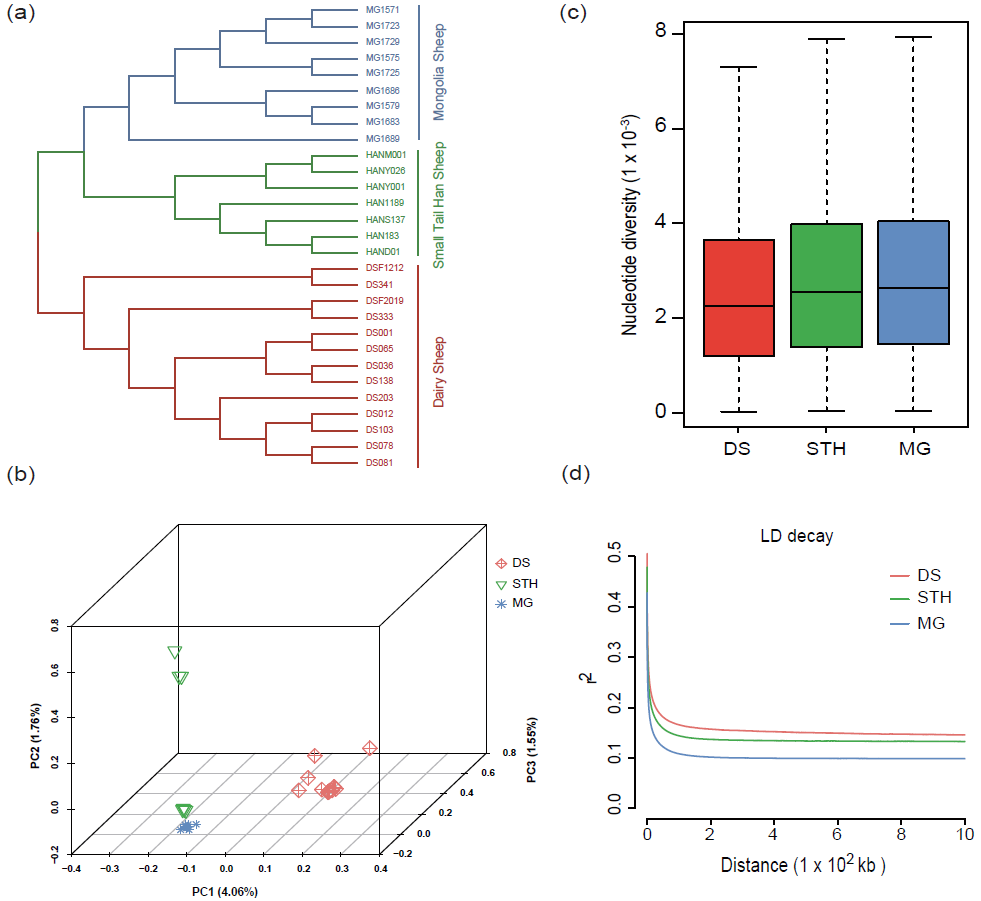


**Fig. S2** Population genetic structure and nucleotide diversity of the sheep populations studied. (a) Phylogenetic tree of all the individuals based on whole-genome SNPs. Diary sheep (DS, red), Small Tail Han sheep (STH, green) and Mongolia sheep (MG, blue) clustered together, respectively. Branch color indicates memberships in different groups. (b) Principal components 1, 2 and 3 from PCA analysis of all the sheep individuals using whole-genome sequences. (c) Boxplots showed nucleotide diversity of DS, STH and MG, calculated in 50 kb sliding window with 25 kb increments across the genome. (d) The decay of linkage disequilibrium (LD) was measured as the squared correlation coefficient by pairwise physical distance in the three sheep populations, with one line per group.


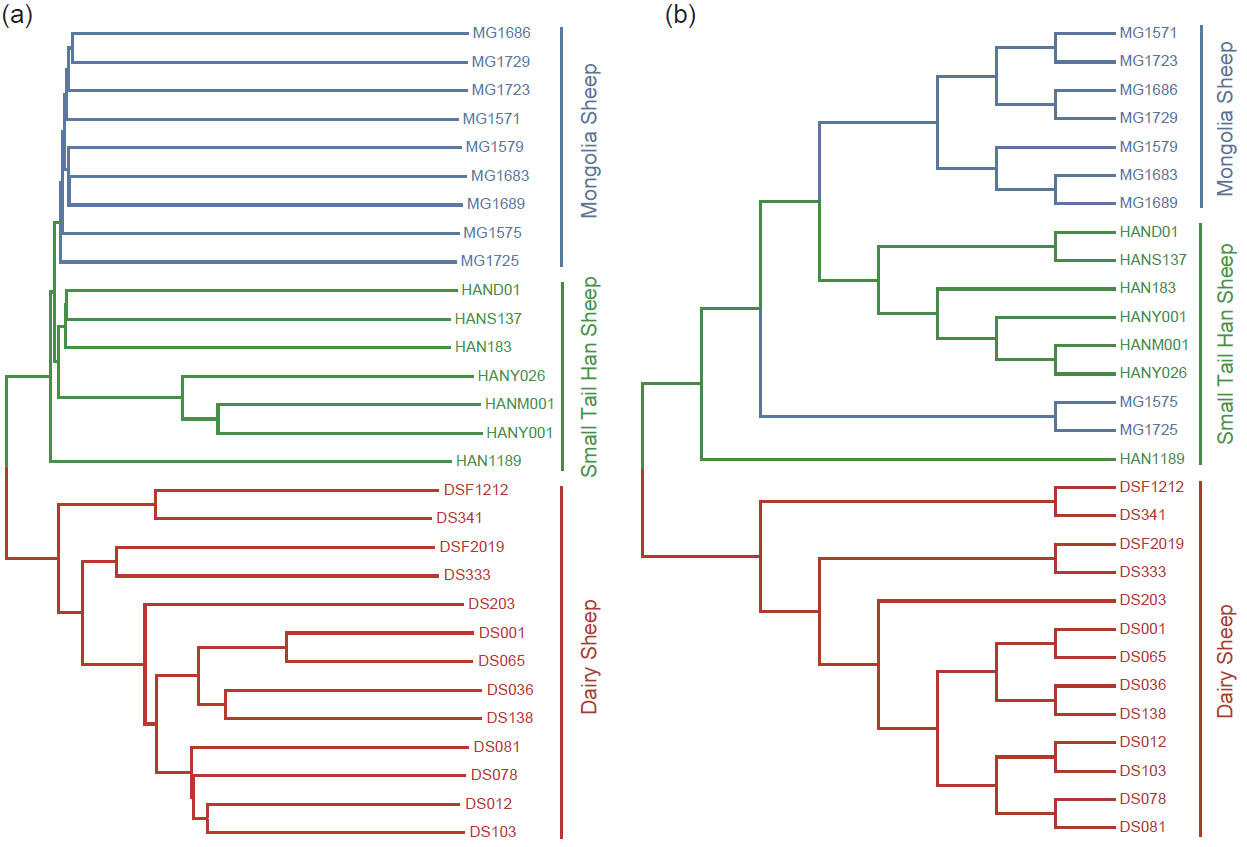


**Fig. S3** Phylogenetic relationship of all the sheep samples constructed by using four-fold degenerate sites. (a)The neighbor-joining tree, sheep individuals clustered into three group. (b) The maximum-likelihood tree, MG1725 and MG1575 are relatively far from other Mongolian sheep.


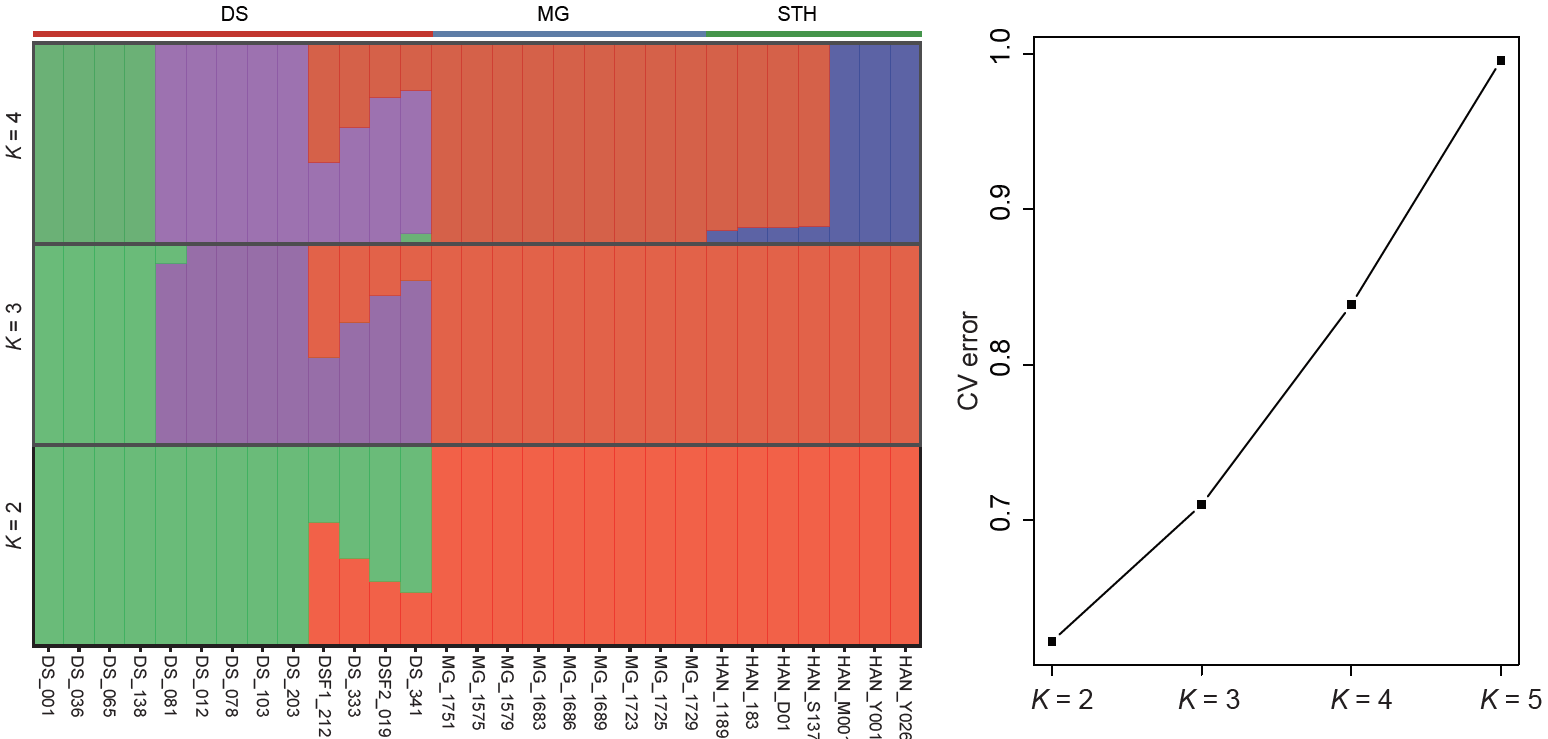


**Fig. S4** Admixture analysis on the subset of 29 sheep individuals. The run with the lowest cross-validation error (out of 20 replicates) is plotted. When *K* = 2 is the best.


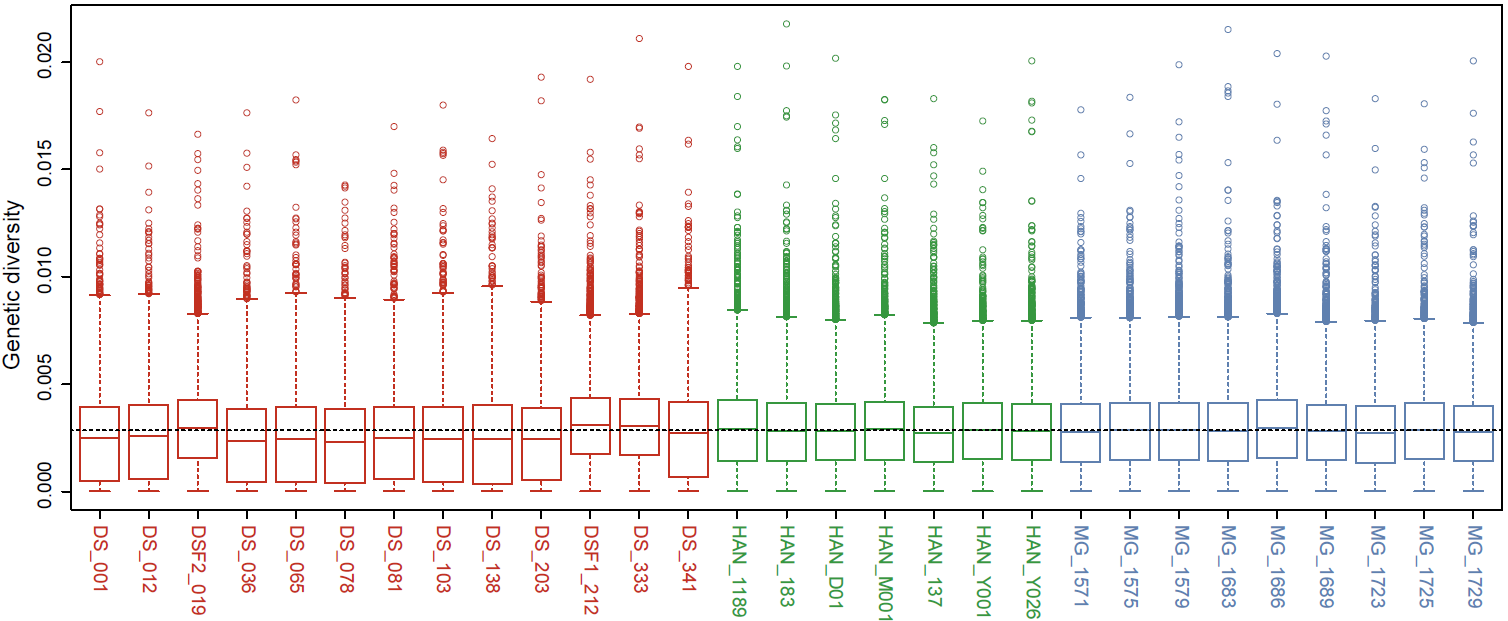


**Fig. S5** Nucleotide diversity of the 29 sheep individuals calculated with the whole-genome genetic variants.


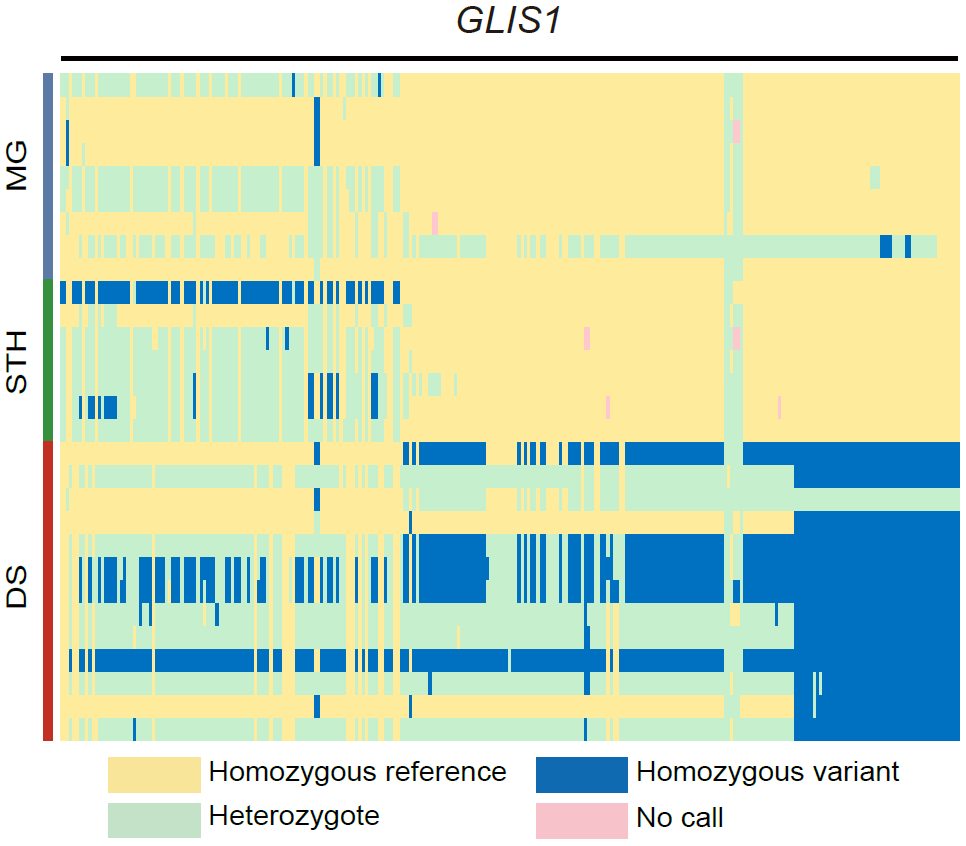


**Fig. S6** Haplotype pattern for the region from 27, 718, 567 bp to 27, 819, 327 bp of chromosome 1. *GLIS1* was encompassed in this region.


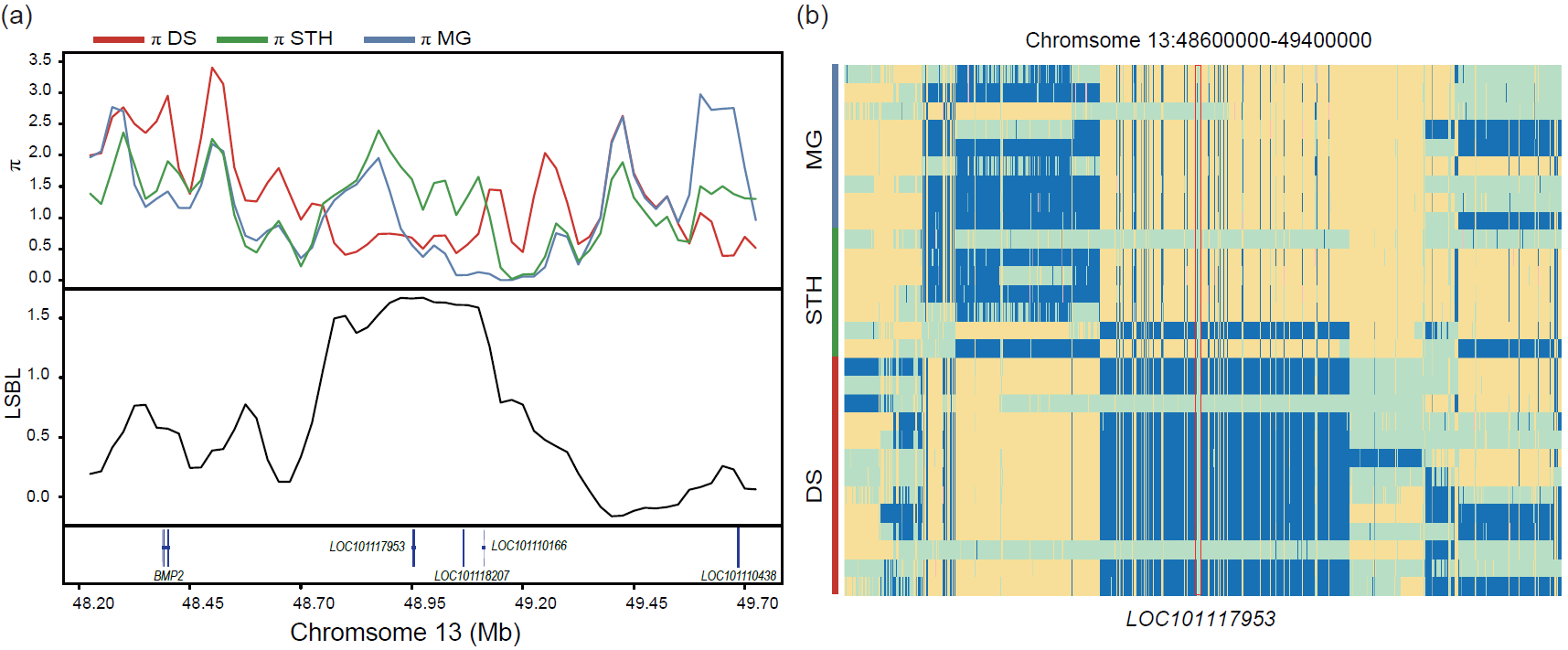


**Fig. S7** A region on chromosome 13 exhibits strong signatures of selective sweep. (a) The comparison of nucleotide diversity and population divergence in DS, STH and MG (50 kb window with 25 kb step increment). (b) Haplotype pattern for the region from 48.60 Mb to 49.40 Mb of chromosome 13. *LOC101117953* was encompassed in this region.


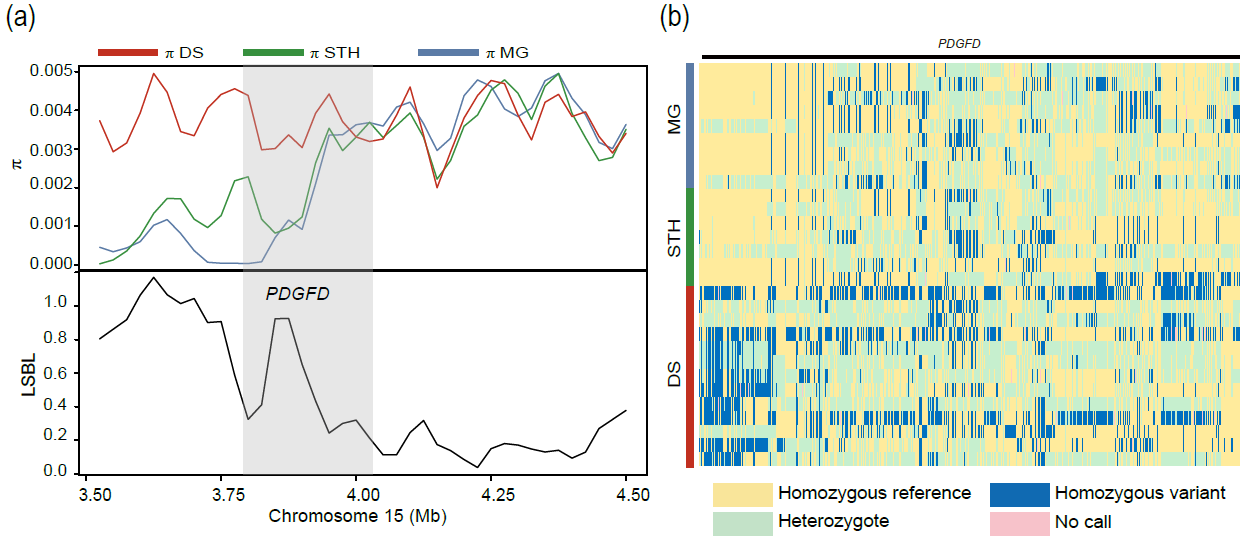


Fig. S8 A region on chromosome 15 exhibits strong signatures of selective sweep.

(a) π and LSBL values around the genomic region on chromosome 15 (from 3.50 Mb to 4.50 Mb) between DS, STH and MG populations. *PDGFD* is located in this genomic sweep region. The red, green and blue lines represent DS, STH and MG population, respectively. (b) Haplotype pattern of *PDGFD* gene among DS, STH and MG.
